# Comparative profiling of the sense and antisense transcriptome of maize lines

**DOI:** 10.1186/gb-2006-7-3-r22

**Published:** 2006-03-13

**Authors:** Jiong Ma, Darren J Morrow, John Fernandes, Virginia Walbot

**Affiliations:** 1 Department of Biological Sciences, Stanford University, Stanford, CA 94305-5020, USA.

## Abstract

**Background:**

There are thousands of maize lines with distinctive normal as well as mutant phenotypes. To determine the validity of comparisons among mutants in different lines, we first address the question of how similar the transcriptomes are in three standard lines at four developmental stages.

**Results:**

Four tissues (leaves, 1 mm anthers, 1.5 mm anthers, pollen) from one hybrid and one inbred maize line were hybridized with the W23 inbred on Agilent oligonucleotide microarrays with 21,000 elements. Tissue-specific gene expression patterns were documented, with leaves having the most tissue-specific transcripts. Haploid pollen expresses about half as many genes as the other samples. High overlap of gene expression was found between leaves and anthers. Anther and pollen transcript expression showed high conservation among the three lines while leaves had more divergence. Antisense transcripts represented about 6 to 14 percent of total transcriptome by tissue type but were similar across lines. Gene Ontology (GO) annotations were assigned and tabulated. Enrichment in GO terms related to cell-cycle functions was found for the identified antisense transcripts. Microarray results were validated via quantitative real-time PCR and by hybridization to a second oligonucleotide microarray platform.

**Conclusion:**

Despite high polymorphisms and structural differences among maize inbred lines, the transcriptomes of the three lines displayed remarkable similarities, especially in both reproductive samples (anther and pollen). We also identified potential stage markers for maize anther development. A large number of antisense transcripts were detected and implicated in important biological functions given the enrichment of particular GO classes.

## Abstract 2

Comparative transcriptome profiling of inbred maize lines demonstrates remarkable similarities and a large number of antisense transcripts.

## Background

Maize geneticists and breeders utilize thousands of inbred and hybrid lines in their research. The diversity of extant lines reflects both the ease of crossing corn (*Zea mays *L.) and the long life of seeds. These lines are derived from hundreds of landraces collected in US farmers' fields and from native Americans beginning in the early 20th century. Lineage records track these materials, the crosses among them, and the inbred lines derived over the past century [1,2]. Phenotypic differences between inbreds can be subtle or dramatic as lines were bred for size, floral morphology, days to flowering, seed constituents, and myriad other traits; distinctive alleles as well as epistatic interactions between loci are the genetic basis for these traits. Differences among lines are notable in genetic analysis when a particular allele, such as a new mutant allele, is introgressed into a range of inbred lines: there can be a striking impact in some lines but a quenching of the expected phenotypes in other lines [3]. Climatic conditions at specific locations also constrain which lines will flourish, reflecting differences in environmental responses. Therefore, it is of great interest to quantify line-specific aspects of gene expression that are the underlying basis for phenotypic variation among inbreds and hybrids and to determine the characteristic patterns of gene expression in specific organs in multiple wild-type lines before examining the impact of mutations on the transcriptome of developing organs.

One complication in defining gene functions in maize is that the species has a tetraploid genome from an event about 11 to 15 mya. The genome retains most of the duplicated chromosomal segments as well as more recently generated duplicated genes [4]. Based on approximately 407,000 public Expressed Sequence Tags, representing parts of gene transcripts, there are 31,375 tentative contigs plus 27,207 singleton sequences totaling approximately 58,582 possible genes (The Institute for Genomic Research (TIGR) Maize Gene Index release 15.0, September 2004), a number likely to shrink to approximately 50,000 with more complete transcript sequencing. Despite the apparent redundancy of genes within this assembly, visible mutants are readily recovered [5]. At present, 6,505 maize loci are defined [6]. Therefore, alleles of many individual genes have distinctive functions in at least one tissue or organ compared to related loci.

A key question that can be addressed with transcriptome profiling is whether lines express the same loci in specific organs and tissues. That is, does the normal phenotype of an organ require that nearly all of the same genes be expressed and in a quantitatively similar manner or can the wild-type condition be achieved despite significant variation in the transcriptome? A related question is how distinctive the progression in gene expression can be during organ development in phenotypically distinctive maize lines. A third question considers whether some organs show more highly conserved patterns of gene expression in diverse lines than other organs, suggesting canalization of the regulatory alleles and of their targets in specifying certain plant parts.

The topic of organ-specific gene expression within one hybrid line was addressed previously by Cho *et al*. [7], who examined 7 organs of maize in a hybrid line composed of 75% inbred K55, 20% W23, and 5% Robertson's Mutator stocks; for roots, leaf blades, and leaf sheaths several developmental stages were examined. A printed cDNA microarray containing approximately 5,600 different genes was used for transcriptome profiling, and the data generated were sufficient to organize a hierarchy of relatedness among the tested organs. As expected, all leaf blade samples clustered together with leaf sheaths as a close sister group; organs associated with reproduction, whether photosynthetic husk leaves or floral organs, clustered together. A major limitation in this study was that cross-hybridization among family members would be expected to obscure many interesting patterns of gene expression; indeed, only 7% of the queried cDNAs showed organ-specific expression, as would be expected if a gene class was required in all the examined organs [7]. The cDNA array format could not determine which member of a recently duplicated gene pair or gene family was expressed in each organ; on a limited scale, suites of oligonucleotide probes printed on the same slide for a few selected gene families showed that short oligonucleotide probes could provide gene-specific data necessary to resolve which family members are expressed in specific patterns [7].

To begin to answer the question of organ-specific expression and to determine the congruence in transcriptomes among lines, a new microarray platform containing *in situ *synthesized 60-mer oligonucleotide probes was employed. A reference design experiment comparing the W23 and A619 derivative lines and W23 and the F1 ND101/W23 hybrid was used with samples from juvenile leaves, mature pollen, and two stages of anther development. In this way, we could examine overlap in gene expression between vegetative, floral, and haploid gametophyte stages as well as determining the similarities between lines. For our validation analysis, both quantitative RT-PCR and hybridization to a second oligonucleotide-based microarray platform were employed.

## Results

### Biological materials and study design

The W23, ND101, and an A619 derivative are Corn Belt Dent varieties, a classification based on origin and seed morphology, but they share no recent common ancestor [1]. They are very similar in gross morphology at all stages of development, but can be distinguished in quantitative traits such as days to flowering, typical seed set, leaf length and width (data not shown). One specific motivation for choosing these lines is that we have begun analyzing male-sterile mutants of maize that are available in these three particular backgrounds. The lines were grown in a common field and four organ types - juvenile leaf blade, 1 mm anther, 1.5 mm anther, and haploid pollen - were recovered for comparison. Mature anthers are sacs composed of four concentric rings of somatic tissue layers; in the middle of each anther hundreds of pre-germinal cells initiate meiosis [8]. Four haploid gametophytes (pollen grains) develop from each meiosis; each pollen grain contains two sperm cells required for the double fertilization characteristic of maize and other flowering plants. Based on Cho *et al*. [7], the expectation was that leaf, anther, and pollen samples would exhibit approximately an equal number of organ-specific transcripts and that the two anther stages would be significantly more similar to each other than to either leaf or pollen. Although these two stages are only one day apart, they are very distinctive developmentally. Within the 1 mm anther, cell divisions are common in the epidermis, in the three internal somatic layers (endothecium next to the epidermis, middle layer, and then tapetum), and in the innermost cell group of pre-germinal cells [9]. Although the somatic cells are already organized into the concentric rings characteristic of a mature anther, cellular specializations are incomplete; the pre-germinal cell population is still expanding, and there is no evidence of pre-meiotic cells (data not shown). At the 1.5 mm stage, each of the cell layers has further differentiated and, based on chromosomal condensation characteristics, meiosis will soon initiate in some of the pre-germinal cells (L Harper and WZ Cande, personal communication).

**Figure 1:**
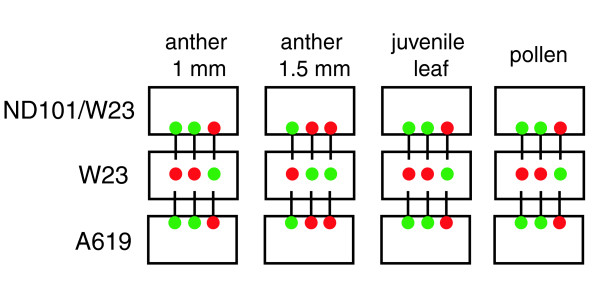
Design of the array experiments. Thirty-six independent biological samples (or pools of staged tissues from the same tassel in the case of the anthers and pollen) were used for eight comparisons. The same aliquot of the W23 sample was used to hybridize to ND101/W23 and A619. Fluorescent dye labeling of each sample is indicated with colors: red for Cy5 and green for Cy3.

Complementary RNAs (cRNAs) from the four tissue stages of A619, hybrid ND101/W23, and inbred W23 were used in two-sample comparisons on a 60-mer *in situ *synthesized array platform (Agilent platform; see Materials and methods for details). As shown in Figure 1, 36 independent biological samples were used for 8 comparisons. The reference design produced six hybridization results for each W23 stage, and there are three biological replicates of the other two lines at each stage. W23 is the standard inbred line for our introgression program and has been previously employed in transcriptome profiling experiments involving leaf tissue [10]; it is the maize line with the most publicly available transcriptome profiling results at the present time.

Because the maize genome has not yet been sequenced, the 22,000 probes for the Agilent arrays were designed from the MaizeGDB December 2003 EST assemblies [11]. Later these probes were mapped onto the TIGR Maize Gene Index assemblies (release 15.0, September 2004). In summary, these probes represent approximately 8,000 sense transcripts, approximately 5,000 antisense transcripts, and approximately 8,000 transcripts with undetermined orientation in this classification. Probes showing significant hybridization were manually analyzed to refine their classification as sense or antisense, and we estimate the array had probes to approximately 13,000 sense transcripts. Note that in the rest of the text, transcripts denote RNA species that were detected on the arrays because they hybridized to one or more oligo probes, either sense or antisense. Generally, the number of hybridized probes is larger than the number of possible transcripts, because there are two or more probes for a subset of genes. When we discuss antisense transcripts, we refer to RNA species that overlap with a known or highly likely cDNA on the reverse strand. The exact length of overlap is not known, but one or more probes to the antisense strand hybridized to the RNA sample with a dye signal above the background threshold. A concern regarding such transcripts might be their generation during cDNA synthesis through fold back self-priming. This will not be a significant problem for the oligo array platform because cRNAs were produced and labeled for hybridizations, although the precise representation of most transcripts was not independently verified in the cRNAs (see Materials and methods).

To identify probes that hybridized, we used an iterative approach and generated statistics from probes that are above background signals in all hybridizations (see Materials and methods for details). Analysis of the final results showed that the thresholds chosen were around the 90th percentile of median signals for the known antisense probes, most of which fail to hybridize with target RNAs, providing a reasonable cross validation of the approach (data not shown). Another benefit of this approach is to remove variances between biological replicates reflecting environmental factors, although this kind of difference is small compared to true line-specific expression differences. For the whole probe set, the correlation coefficients of the raw dye median intensities between each pair of biological replicate are mostly between 0.95 and 0.98, even when they were labeled with different dyes and presumably dye bias could have an effect. This is comparable to technical variances as assessed by duplicated probes on the arrays and both can be removed effectively by our approach.

### Distinctive patterns of gene expression in organs and by genetic background

As shown in Table 1, approximately 5,700 transcripts showed a positive hybridization signal in each anther and juvenile leaf sample. In contrast, about half as many transcript types were detected in pollen samples. Because the probe designs were based on EST data, they are weighted toward more highly expressed genes, and we therefore consider it significant that specific probes fail to hybridize with certain tissue samples. The total transcriptome of each sample is likely to be considerably larger than reported here, because the array platform contains probes to detect only about 25% of the expected gene transcripts of maize [12].

**Figure 2:**
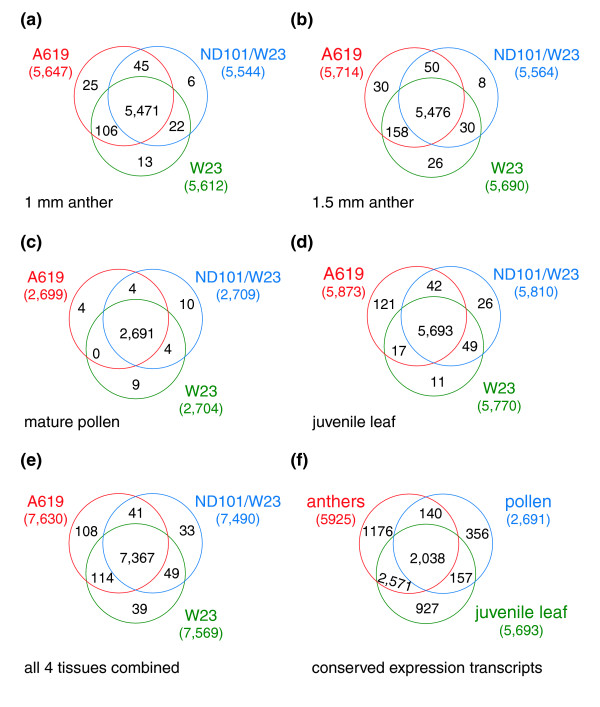
Venn diagrams of transcript representation. **(a-d) **Tissue analysis: the transcripts shared among the three genotypes at the four developmental stages examined are depicted. **(e) **Overlap between transcripts pooled for each line. **(f) **Overlap between conserved transcripts among the three lines for each tissue type. Transcripts hybridized in either of the two anther samples were combined to form a single collection.

In terms of gene expression patterns, the juvenile leaves had the most distinctive transcriptome, with approximately 18% tissue-specific transcripts in A619, ND101/W23 and W23 compared to anthers or pollen. Pollen, representing a 10 to 20 minute interval during pollen shed from the anther, was the most discrete stage collected in terms of temporal development; pollen contained approximately 14% sample-specific transcripts in the three lines examined. Anther stages, which differ by one or two days of development, exhibited approximately 5% stage-specific transcripts at the 1 mm size and approximately 4% stage-specific transcripts at the 1.5 mm stage. If the anther data are combined and treated as one stage for comparison to pollen and juvenile leaf, anther-specific transcripts increase to 20% (Figure 2f), and collectively exceed the juvenile leaves.

Because a two-color hybridization protocol was employed in which each A619 or hybrid ND101/W23 sample was compared to W23, it was also feasible to define differentially expressed genes in the paired tests. A619 showed more differences compared to W23 than did the F1 hybrid of ND101 with W23; there were approximately 300 differentially expressed genes in each anther stage and in leaf in the A619-W23 comparison and fewer than 100 for pollen. The number of differences in the W23-ND101/W23 comparison was about half of the A619 differences in the anther samples but very similar for the other two tissues. Although parentage should be highly predictive of gene expression patterns, and it would therefore be logical to expect A619 to be more distinctive than the F1 hybrid, hybrid vigor is an important consideration. This phenomenon was discovered in maize at the beginning of the 20th century [13]; after inbreeding depresses plant yield and growth, combination with another inbred line typically yields an F1 hybrid far superior to either parent, suggesting significant changes in gene expression. Nonetheless, for the lines examined here, the ND101/W23 hybrid is more similar to W23 than the heterologous A619 line.

The complete results from the analysis of the common and unique transcript types in each genotype as well as across tissues are shown using Venn diagrams in Figure 2. Pollen and both anther stages have highly conserved transcriptome patterns, because fewer than 1% (both pollen and 1 mm anther) or about 1% (1.5 mm anther) of the transcripts are uniquely expressed in one line compared to the total shared in all 3 genotypes. In contrast, approximately 3% of the transcripts are line-specific in juvenile leaves. A global genotype analysis was conducted (Figure 2e) in which all four tissue samples were combined within each genotype. Comparing the three genotypes on this basis again highlights that A619 is the most distinctive, while W23 and the hybrid ND101/W23 are much closer in transcriptome pattern. In the global tissue analysis (Figure 2f), only transcripts that are expressed in all 3 lines (7,367 in total) were considered, and the 2 anther stages were treated as a single tissue type. There were 2,038 transcript types in common among the three biological sample types, the beginning of an enumeration of constitutively expressed or 'housekeeping' genes for maize. In the global assessment it is also clear that juvenile leaf and anthers share many transcripts in common (2,571), twice the number that each organ uniquely expressed. Pollen and the other two tissue types share approximately 150 transcripts each, about 11% of the 2,691 pollen transcripts found, indicating that although fewer transcripts are expressed than in other tissues examined (compare to 5,925 for anthers and 5,693 for leaf), there is a distinctive suite of transcripts present in pollen (>13% unique transcripts).

**Figure 3:**
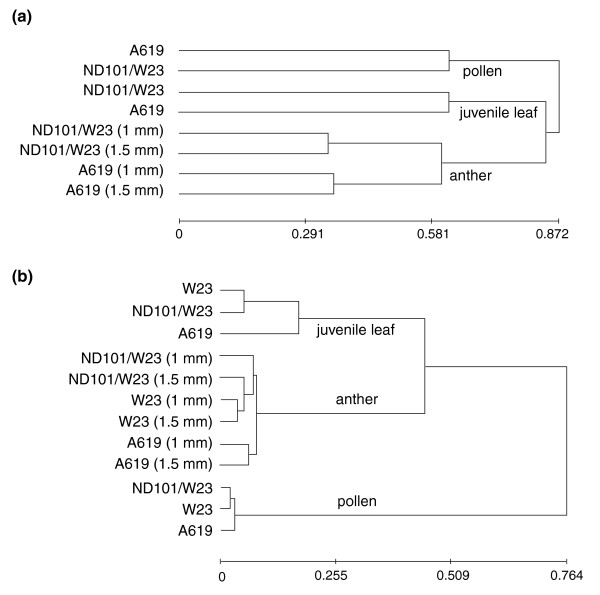
Average linkage clustering trees based on correlation measure based distance (uncentered). Distances are calculated from **(a) **log2 ratios of either A619 versus W23 or ND101/W23 versus W23 and **(b) **normalized log2 absolute intensities. See Materials and methods for details.

An alternative method of assessing the relatedness among the samples is to construct clustering trees as shown in Figure 3. In Figure 3a, the tree is based on the log2 ratios of A619 and ND101/W23 transcripts each in comparison to the W23 inbred line. Pollen is the most distinctive sample type, while leaves and anthers cluster together. In this diagram, it is clear that the 1.0 and 1.5 mm anther stages of each genotype share more in common than the length-based stage of one genotype shares with the comparable length sample from the second genotype. Although length is a reliable classification method in the sense that anthers elongate and enlarge progressively throughout development, the precise developmental stage in terms of transcriptome is clearly complicated by genotype differences and unavoidable inaccuracies in sample collection. This conclusion is reinforced when the normalized log2 absolute intensities from all three genotypes are used for constructing the tree (Figure 3b). The hierarchy of relatedness is similar to the global tissue analysis in Figure 2 in which pollen is the most distinctive and juvenile leaves cluster (distantly) with the anther samples.

**Figure 4:**
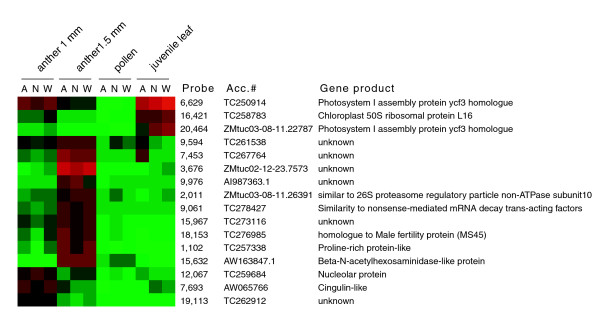
Potential marker genes for the two anther stages based on similar expression values in all three lines. The coloring is based on the log2 values of absolute dye intensities normalized to the median value of all hybridized probes in a given tissue sample. The high and low expression probes are shown in red and green, respectively: the higher the absolute value of the hybridization signals deviates from the median, the brighter the color. A, A619; N, ND101/W23 hybrid; W, W23.

These data also greatly extend the list of presumptive stage-specific genes in maize, and because 60-mer oligonucleotide probes were used, an assignment of a specific locus is usually secure. Lists of stage-specific genes that are expressed in all three lines are in Additional data files 1, 2, 3, 4. Figure 4 shows some of the potential markers identified. The expression values are log2 of absolute dye signals normalized against the median of all the hybridized probes in a given sample; therefore, they are comparable between lines and tissues. The accession numbers are from MaizeGDB [11], TIGR [14], or NCBI GenBank. It is quite striking that some of the photosynthesis genes, including two Photosystem I assembly protein *ycf3 *homologs (TC250914 and ZMtuc03-08-11.22787) and a chloroplast 50S ribosomal protein L16 (TC258783), are highly expressed not only in the leaf as expected but also in the early anther stage (1 mm stage). These transcripts decrease at the next stage of anther development just prior to meiosis, although they were still detectable. A cigulin-like gene (AW065766), a nucleolar gene (TC259684), and an unknown gene (TC262912) are potentially markers for the 1 mm anther stage (Figure 4). There are also several good marker candidates for the more advanced 1.5 mm anther stage, including a putative nonsense-mediated mRNA decay trans-acting factor (TC278427) and a male fertility protein (TC276985), annotated as a strictosidine synthase, a key enzyme in alkaloid biosynthesis. TC276985 turned out to be the *ms45 *gene; the gene product was found to be localized to the tapetum and expressed maximally during the early vacuolate microspore stage of anther development [15]. This literature report validates one of the stage markers and increases confidence in the additional proposed markers.

### Enrichment of Gene Ontology classes

To gain further insight into processes that change during anther development, we analyzed the functional interactions between gene classes in the transcriptomes under study. There is currently no official release of Gene Ontology (GO) annotations for maize genes; therefore, we used the program Blast2GO [16] to assign GO terms based on protein sequence similarities and associations. We also downloaded GO annotations for the TIGR Maize Gene Index sequences, if available. Subsequently, the Gossip program was used to find statistically significant enrichment of certain GO terms in the test group against a reference group [17]. For the expressed sequences, 5,338 were successfully assigned at least one GO term. Each test group is a specific class of transcripts, for example, anther-specific transcripts. For this test group, the reference group was the remaining GO-annotated transcripts that do not belong to the test group; these test and reference groups were compared to search for significant enrichment (Table 2).

In general, the GO analysis displayed very consistent patterns in accordance with already well-known functions of a given tissue type (Table 2). Leaf-specific genes are abundant with terms related to the plastid (GO:9536) and the key step in photosynthesis, oxygen binding (GO:19825). Over-represented GO terms for anther-specific genes include cyclin-dependent protein kinase regulator activity (GO:16538), DNA replication initiation (GO:6270), and a great number of genes involved in nucleic acid metabolism (GO:6139). On the other hand, pollen-specific genes are enriched in pectin esterase activity (GO:30599), a gene family that has been shown to function specifically late in pollen development [18], hydrolase activity (GO:16787), secretory pathway and secretion (GO:46903), transport (GO:6810), cell wall modification and cytoskeleton activities, among many other cellular functionalities that underlie a series of biological processes during pollen maturation. Not surprisingly, the ubiquitous endomembrane system (GO:12505) is represented in all tissue types. These results indirectly confirmed the utility of mining the GO data structure by this method. When we tested the differentially expressed gene groups, none showed any significant over-representation except in the comparison of W23 samples to the ND101/W23 pollen and juvenile leaf (Table 2). Interestingly, the GO analysis showed that the differentially expressed genes in the ND101/W23 hybrid pollen sample are enriched in negative regulators of apoptosis and programmed cell death (GO:43067, GO:6916). In the leaf sample, genes involved in oxidoreductase activity (GO:16491) and chloroplast (GO:9507) functions are differentially regulated. The functional significance of these gene regulations to the plant and their possible connection to the hybrid genomic background remain to be tested.

### Antisense transcripts detected for many genes

Natural antisense transcripts (NATs) have been identified experimentally and predicted computationally from many organisms, including human, mouse, yeast, fruit fly, and *Arabidopsis *[19-23]. By definition, NATs contain sequences complementary to the sense transcripts of protein-coding genes. They may be transcribed *in cis *from the reverse strand (called *cis*-NAT) or *in trans *from separate loci (called *trans*-NAT). In eukaryotes, the majority of NATs are of the *cis *type. Unexpectedly, NATs are common: up to 20% of human genes have a NAT. Furthermore, many NATs are conserved, implying regulatory functions for these transcripts in eukaryotic gene expression [22,24,25]. To address the question of what fraction of maize genes might be regulated through an antisense transcript, the array platform was constructed to contain approximately 5,000 probes to detect the antisense strand of gene models constructed from EST assemblies; in some cases more than one 60-mer antisense oligo was designed per gene.

In Table 3, the percentages in the antisense category versus the total transcripts detected (Table 1) are shown for all four developmental stages in the three genotypes. The percentages of antisense transcripts are highly consistent within each tissue type but there is substantial diversity among the tissues. In detail, the three tissue samples with approximately 5,700 hybridizing probes *in toto *exhibited different percentages of antisense transcripts: 11% for juvenile leaf, 6.5% for 1 mm anther, and 7.5% for 1.5 mm anther. Even more strikingly, 14.3% of the pollen transcriptome consists of antisense transcripts. These results indicate that a surprisingly large fraction of maize genes are represented by a detectable antisense transcript. As with sense transcripts, there is considerable overlap in the tissue distribution of the antisense transcripts, although very consistent percentages of the transcripts were tissue-specific. Strikingly, more than one-third of the antisense transcripts in juvenile leaves are found only in that tissue source in each genotype, with about 10% stage-specific antisense transcript present in the pollen and 1.5 mm anthers while only about 4% of the detected antisense transcripts in 1 mm anther were found only in that stage (Table 3).

**Figure 5:**
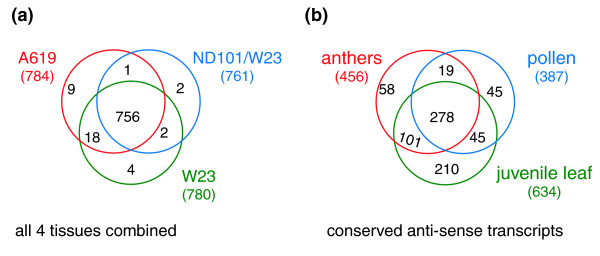
Distribution of antisense transcripts. **(a) **Global analysis of antisense transcripts in all four tissue samples combined. **(b) **Tissue analysis of the 756 antisense transcripts conserved in all three lines, after pooling data for the two anther stages into one collection.

The distribution patterns of these detected antisense transcripts among the three lines are shown in a Venn diagram (Figure 5a). These patterns are extremely similar to the distribution of overall (both sense and antisense) transcripts; only about 2% of the antisense transcripts are unique to one line, and more than 95% are shared among the three lines. This striking consistency makes it likely that these antisense transcripts are biologically functional rather than array artifacts. In Figure 5b, we then combined the two anther stages and considered only the 756 antisense transcripts (Figure 5a) shared among all three lines. Compared to the global distribution, there are both more tissue-specific (41% ((58 + 45 + 210)/756) compared to 33%) and more common (shared among all 3 tissue types; 37% compared to 28%) antisense transcripts. Furthermore, the percentages of antisense transcripts versus the corresponding total transcript category (Figure 2f) are vastly disparate. Specifically, only 5% of the anther-specific transcripts (58 out of 1,176) are categorized as antisense, compared to 13% of pollen-specific and 23% of leaf-specific transcripts. Therefore, the transcriptomes of both pollen and leaf contain more tissue-specific antisense species than do anthers; anthers express mainly common antisense transcripts. An outcome of considering the antisense transcripts separately is that approximately 14% (278 out of 2,038) of the total common transcripts shared among all 3 tissue types and 14% of the transcripts shared between pollen and anthers are antisense. In pair-wise comparisons, only 4% of the transcripts shared between leaf and anthers are antisense, in sharp contrast to the transcripts shared between only pollen and leaf, 29% of which are likely to be antisense.

Because NATs are often discussed in the context of the corresponding sense transcripts, we identified 1,063 potential transcripts on the array that are represented by at least one pair of sense-antisense probes. Considering all the hybridization data, for 136 such pairs both probes hybridized, indicative of both sense and antisense transcripts in the RNA samples (see Additional data file 5), for 665 only sense probes hybridized, and for 41 only antisense probes hybridized (data not shown).

**Figure 6:**
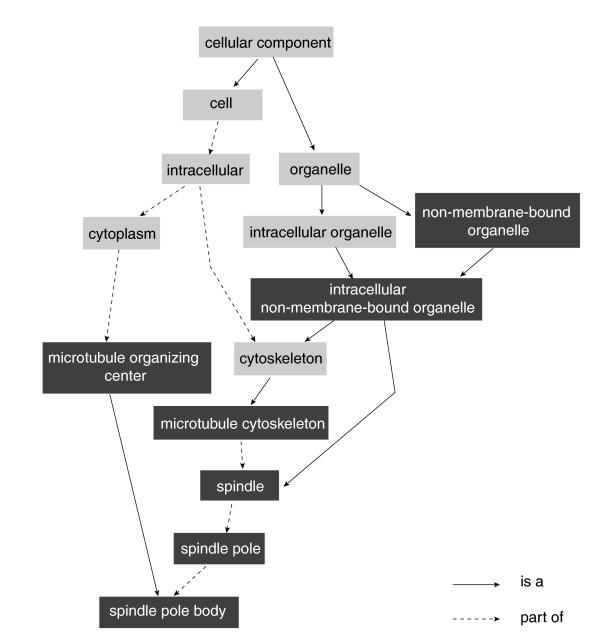
GO analysis of detected antisense transcripts. Dark squares indicate statistically significant over-representation of GO terms among 732 antisense transcripts with assigned GO annotations (see text and Table 4 for details; a list of expressed 'spindle pole' and related genes are given in Additional data file 7).

A GO classification was conducted to determine the representation of antisense transcripts detected by the arrays. We were able to assign GO annotations to 732 transcripts that showed above-background hybridizations to at least one antisense probe. When comparing the represented genes with the whole set of hybridized transcripts with GO terms assigned, two classes dominated the GO classifications (Table 4). One belongs to organismal physiological processes (GO:50874); these are processes pertinent to the organism functions above the cellular level and include the integrated processes of tissues and organs. Other enriched terms include perception of light and photosynthetic electron transport. A large fraction of these are 'organismal physiological processes'. Another unexpected finding was the over-representation in the antisense group of cell cycle related transcripts (Table 4), especially genes with homologies to spindle pole and spindle body related genes in other organisms, although plants lack a spindle pole during mitosis. There are 21 genes in the cell cycle related sub-classes that have detectable antisense transcripts, and each of the three tissue types expresses at least 14 of them. Three of the 21 had transcripts in both sense and antisense orientation. In addition, fifty-seven genes in these sub-classes had only sense probes on the arrays. The relationships between these GO terms are diagramed in Figure 6. The prevalence of antisense transcripts for genes involved in such critical cellular processes will motivate a more detailed study of the true function of these antisense transcripts.

### Validation of microarray data

**Figure 7:**
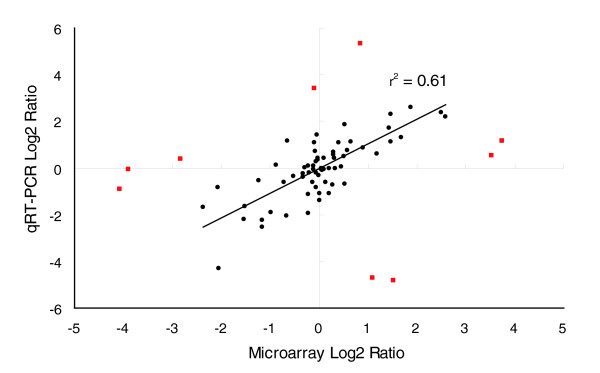
Correspondence of qRT-PCR and array hybridization results. Outliers that were excluded from the regression analysis are indicated in red. Primer sequences, putative gene products for the probes, and expression values are given in Additional data file 6.

Two approaches were employed to validate the results of the array hybridization experiments. Quantitative real-time PCR (qRT-PCR), which has been widely used for selective verification of array results, was employed for 23 examples of genes expressed in all or a subset of specific tissue types. The expression levels of these genes cover a wide spectrum so that we could compare the resolution and relative accuracy of the two techniques. We picked two internal standards for each tissue stage based on published results [26] or their known stable expression in a given tissue in maize or other plant organisms, for example the heat shock 70 kDa protein (see Additional data file 6). Again we used the four stages from W23 and ND101/W23 with which we did the microarrays, and two to four biological replicates of independent biological samples were tested for this panel of genes. The results were averaged to remove both biological variances caused by environmental factors and technical variances. As shown in Figure 7, there is a good correspondence (r^2 ^= 0.61 when excluding 9 apparent outliers) between the qRT-PCR log2 ratios and the array log2 ratios (ND101/W23 compared to W23). Of the 18 transcripts whose expressions were not detected by the arrays for any given stage (not plotted in Figure 7; see Additional data file 6), 14 were not detected by qRT-PCR either, further confirming the correspondence between the two methods. It also provided supporting evidence for our assessment of a gene transcript being 'present' or 'absent' solely based on array hybridization intensities. The 'outliers' were most likely caused by cross hybridizations from highly similar gene family members in the array results. Any further analysis of these probes/genes will have to wait until the complete genome sequence becomes available.

**Figure 8:**
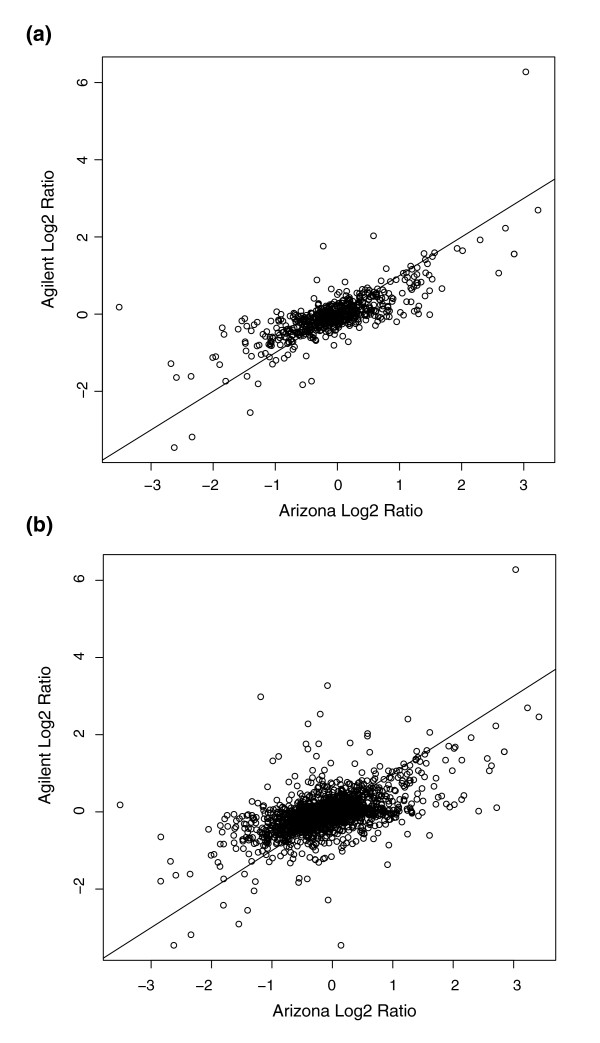
Comparison of hybridization results between the 60-mer Agilent and 70-mer University of Arizona (slide A) array platforms. Probes from the two sets designed for the same gene/EST sequences were divided into two groups: **(a) **within 30 bases of each other; and **(b) **more than 30 bases apart. Data in the plots were from hybridizations using the same 1.5 mm anther tissue samples and the same design as in Figure 1. A similar analysis was performed for slide B of the Arizona set (data not shown).

A more complete validation was performed by repeating the A619/W23 hybridizations with the identical anther RNA samples utilized on the 60-mer *in situ *synthesized array to a second microarray platform consisting of 70-mer spotted oligonucleotides on 2 slides. Slide A of this platform contained 29,270 maize gene oligos plus a number of controls, and thus has more than twice as many sense gene probes as the Agilent platform. There are 3,568 genes represented on both platforms in the same orientation: 1,155 of these partially overlap (that is, they were designed to the same region of the gene) and 2,413 of the probes are designed to different parts of the same gene model. As shown in Figure 8a, for the probes designed to the same region of the gene (within 30 bases), there is a very good correspondence (r^2 ^= 0.77) between the log2 intensity ratios. These data cross-validate the two platforms as reporting the same transcriptome information for many genes. The correspondence is good for probes that hybridize to different parts of a gene transcript (Figure 8b; r^2 ^= 0.56). Slide B of the second platform has 27,339 maize gene oligos. Of these, 4,092 match genes represented on the Agilent array with 1,294 designed to the same gene region and 2,798 oligos in different gene regions. Similar correlation coefficients were observed for the log2 ratios for the two categories of oligos (r^2 ^= 0.75 and 0.44, respectively; data not shown).

## Discussion

Maize has been shown to display considerable genomic heterogeneity and non-colinearity between inbred lines. These differences reflect mostly insertions of many transposable elements and translocation of individual loci from one chromosome to another, a process likely mediated by transposons [27,28]. Brunner *et al*. [28] recently examined more than 2.8 Mb of maize syntenic chromosomal regions in two inbred lines and found more than one-third of the loci are absent in one inbred. Therefore, a key question is whether lines express the same loci in specific organs and tissues even when loci are in a new chromosomal context. Our results showed that despite the many likely instances of genomic non-colinearity in the 3 lines examined, they share more than 95% of their transcripts (Figure 2e). Furthermore, quantitatively about 95% of the transcriptomes are expressed at comparable levels between the two inbred lines W23 and A619; there is an even greater congruence between W23 and the hybrid ND101/W23 (Table 1; Figure 3b). Thus, although there is high nucleotide polymorphism in maize genes, the 60-mer and 70-mer probes are likely to hybridize well across lines. We conclude, therefore, that the non-colinearity observed for maize inbred lines seems to have little effect on the transcriptome in three major organs - leaf, anther and pollen. A related question concerns development *per se*: does the normal phenotype of an organ require that nearly all of the same genes be expressed and in a quantitatively similar manner, or can it be achieved with significant variation in the transcriptome? Because of the very high overlap in expression among the lines at each stage, the normal phenotypes are achieved with near-identical patterns of gene expression. The differences identified, although relatively few in number, will be important in further studies to relate the quantitative phenotypic differences distinguishing each line to the expression of specific transcripts.

It should be cautioned that transcriptome analysis using microarrays is plagued by two universal caveats: cross hybridization and the limitation in detection resolution. It may be even more severe for the maize genome given the high polymorphisms between inbred lines and the prevalence of duplicated genes. The problem of cross hybridization can be circumvented by careful probe design. Because the maize genome has not been completely sequenced, the probes on our arrays may cross hybridize with yet undefined gene transcripts. Therefore, our conclusions attest mainly to the congruence of overall gene expression in the three genetic backgrounds. In considering the second problem, statistically insignificant expression differences may be biologically significant and cause quantitative phenotypic differences. In recent years, efforts have been made to map gene expression quantitative trait loci (eQTL) and link them with classic quantitative traits. Both *cis *and *trans*-acting eQTLs have been identified for regulatory loci in yeast, maize, *Arabidopsis*, human and mice [29-32]. Thus combining microarray and eQTL analyses has proven to be more powerful in elucidating genetic control of gene expression in maize and other organisms.

A third question concerns how distinctive gene expression is among the organs examined. If we look only at the transcripts that are expressed in all three lines (thus with a high confidence of their expression), more than one-third of the transcripts for any single tissue are shared among all three tissues, and for pollen the frequency increases to more than three-quarters (Figure 2f). This might reflect the bias of the probes towards highly to moderately expressed genes. Nonetheless, compared to the work of Cho *et al*. [7] showing that only 7% of transcripts were tissue-specific after hybridization to a cDNA array platform, we find that one-third of the combined transcripts are tissue-specific. Even more striking is the large number of transcripts shared between leaf and anther, including several photosynthesis genes that are expressed highly in early anther (Figures 2f and 4). This certainly provides evidence to strengthen the model that anthers and other floral organs are modified leaves.

A fourth question considers whether some organs show more highly conserved patterns of gene expression in diverse lines, which may indicate canalization of the regulatory genes and of their targets in specifying certain plant parts. From the transcriptome analysis, reproductive tissues, represented by anther and pollen, express a more conserved transcriptome than vegetative tissues, represented by leaf. Both A619 and ND101/W23 had much more line-unique transcripts that are also specific for leaf than for either pollen or anther (Figure 2a–d). As for expression levels, both lines also showed more differentially expressed transcripts in the leaf than in the anthers (Table 1). The conservation of gene expression patterns during anther development and pollen function may be important to insure reproductive success.

Because ND101/W23 is a hybrid and much more robust than W23, one interesting question to ask is whether heterosis (hybrid vigor) is determined by drastic transcriptome changes compared to a parental line. Fu and Dooner [33] proposed that complementation of weak, line-specific alleles could partially contribute to hybrid vigor. However, accumulating evidence suggests that dosage-dependent, non-additive gene expression may play a bigger role [34]; that is, epistatic interactions among new combinations of alleles result in the significant phenotypic differences between many hybrids and their parents. For example, Song and Messing [35] found unexpected differences in the expression of shared and line-specific genes in reciprocal hybrids of two maize inbred lines. Our results demonstrate that the ND101/W23 hybrid is actually very close in gene expression to W23 in every tissue sample tested (Figure 3). It does share about the same number of common transcripts with either W23 or A619 (Figure 2e), however, suggesting an unbiased expression of line-specific genes. Given the lack of data from reciprocal hybrid lines between W23 and ND101, and also from the parental ND101 inbred, we could only speculate on this important question in maize genetics.

Natural antisense transcripts have been implicated in the regulation of a number of biological processes, including RNA interference, translation regulation, alternative splicing, genomic imprinting, and RNA editing. However, very few NATs have been experimentally analyzed, and the exact roles of the large number of NATs in seemingly every eukaryotic genome analyzed so far remain elusive [19-25]. Nonetheless, even though their possible functions in the maize genome are largely unknown, the diversity of antisense transcripts discovered in this study indicates that this class of RNAs is likely to play important roles in maize development and physiology.

This report also provided a good cross validation between two array platforms, each having specific strengths. The Agilent platform displayed superb hybridization images and a very consistent low background. On the other hand, the University of Arizona platform provided many more probes and hence much wider coverage of the maize transcriptome.

## Conclusion

Despite the phenotypic and genotypic diversity of maize, transcriptome profiling indicates that the three lines tested share remarkable similarities in gene expression patterns across diverse tissue types, especially in both reproductive tissues (anther and pollen). Our ultimate goal is to define the genetic basis for anther morphology and the functions of cells within this floral organ. We are using diverse male-sterile mutants that affect the differentiation of anther cell types at specific stages to define organ ontogeny. As plants lack a germ line, it is of particular interest to define the mechanisms underlying pre-germinal fate determination, which requires that somatic cells become competent to initiate meiosis. More than 400 male-sterile mutations are available, but they are in diverse genetic backgrounds. Because only two or three generations of corn can be grown annually, introgression to the status of a near-isogenic inbred line can require years. We were therefore motivated to determine the extent of line-specific gene expression in anthers that could confound comparisons between different male-sterile mutants and a reference male-fertile line into which all the mutants would eventually be introgressed. Our results show that despite a congruent transcriptome observed across the different genetic backgrounds, the number of differentially expressed genes is still considerable. Therefore, any mutant to wild-type comparisons will be carried out using sterile and fertile siblings in the same family to circumvent the problem.

## Materials and methods

### Biological materials and tissue collection

The ND101 line was supplied by P Bedinger and the A619 derivative by W Sheridan. The W23 line carrying the *bz2 *mutation (lack of anthocyanin accumulation) is maintained in the Walbot laboratory by self-pollination. These materials were grown at Stanford University in the summer of 2003 and phenotypes were quantified (data not shown); the lines were propagated by self-pollination of male-fertile individuals and by crosses of W23 as pollen parent onto the ND101 male-fertile individuals. For collection of tissues, the resulting lines were grown in summer 2004 at Stanford University; leaf and pollen samples were collected in the field, transferred to a labeled plastic tube, and immediately frozen in liquid nitrogen. Multiple biological samples from fully expanded juvenile leaves (leaves 8, 9, 10 in these lines) on different plants were harvested. At flowering, tassels were bagged to collect pollen shed from exerted anthers, which was then sieved to remove extraneous debris. Pollen from the same individual was pooled to make one biological sample. Multiple biological samples were collected over a period of several days for each line. Anthers must be dissected from developing flowers; to do this, plants of approximately the correct stage were identified in the field on the basis of tassel size, and the entire plant was harvested by cutting near ground level with a knife. The harvested plants of each line were kept in separate buckets of water in an air-conditioned field laboratory. A maize tassel contains hundreds of flowers, borne in pairs called the upper and lower floret. Each floret contains three anthers. Because the upper florets mature more quickly than the lower florets, and the two floret types exhibit some transcriptome differences at the 2 mm stage of development (D Skibbe, personal communication), dissection was restricted to upper florets. Anthers were dissected into 2.0 ml microfuge tubes containing liquid nitrogen; the tubes were supported in a styrofoam pad and periodically refilled with liquid nitrogen. For 1 mm anthers, a sample of several hundred anthers was collected for each genotype, typically from just one tassel. Approximately 100 anthers at the 1.5 mm stage were sufficient for an RNA extraction suitable for microarray and RT-PCR analysis. Up to 15 replicate samples were obtained.

### Array design and analysis - Agilent platform

Agilent Technologies microarrays are built using phosphoramidite chemistry to synthesize 60-mers *in situ *on glass slides [36]. There are 322 internal positive and 314 negative controls on each maize array. Maize probes were designed from the December 2003 maize EST assembly of MaizeGDB [11]. The 21,939 maize probes represent 21,782 unique probes, with 157 probes duplicated. Hybridizations for duplicated probes for the 8 experiments were highly correlated as assessed from correlations between median signal intensities (r^2 ^= 0.97 for both dyes; data not shown) and between log2 ratios of the signals (r^2 ^= 0.94; data not shown). Oligonucleotide sequences, gene identities and both raw and normalized hybridization intensities for each probe can be downloaded from our array data submitted to the Gene Expression Omnibus database [37].

To identify unique genes or transcripts, we mapped the probe set to the TIGR Maize Gene Index (release 15.0, September 2004) [14]. The TIGR dataset provides annotations for each Tentative Contig assembly based on protein similarity search results and EST sequence orientation information (for example, the presence of a poly-A tail). The assembly will be annotated as 'coding strand' if there is strong supporting evidence. By the stringent criterion of at most 2 mismatches over an alignment length of 60 nucleotides (the full probe length), the probes were found to represent approximately 21,132 unique transcripts (either sense or antisense; see below).

#### Identification of antisense probes

We used a combination of two independent approaches to identify antisense probes. First to avoid assembly errors, the probes were mapped back to their original EST sequences (downloaded from NCBI), and the corresponding EST sequences (with an average length of 555 base-pairs) were subjected to a BLASTX similarity search against a plant protein database extracted from UniProt (the December 2004 dump). The following criteria were used: first, the top hit must be from peptide translated from the reverse strand of the EST and the BLAST score >80; and second, if there is also a hit(s) from the sense strand, its BLAST score must be below 50 and the top score must be over 100 (for a reverse hit). The BLASTX results were cross-validated by mapping the probes to the TIGR Maize Gene Index dataset, which provides additional information on the orientation of the TC sequences. A probe is annotated as 'antisense' if both the BLASTX results and TIGR Maize Gene Index evidence showed it to hybridize to the reverse strand of a coding sequence. A total of 5,075 probes were identified as antisense probes. To further confirm this probe set, we randomly picked 100 probes and manually verified that they were antisense probes given available information on the maize transcriptome.

#### RNA extraction, target cRNA preparation and array hybridization

Total RNA was extracted from 30 to 60 mg of frozen tissues at each developmental stage using the RNeasy Plant Kit (Qiagen, Valencia, CA, USA) and subjected to DNase I (Invitrogen, Carlsbad, CA, USA) or Turbo DNase treatment (Ambion, Austin, TX, USA) and a second round of RNeasy column purification (Qiagen). The yield and RNA purity were determined spectrophotometrically with a SpectraMax 250 plate spectrophotometer (Molecular Devices, Sunnyvale, CA, USA) and verified by agarose gel analysis. Target cRNA was prepared and labeled with either Cy-3 or Cy-5 dye (PerkinElmer, Boston, MA, USA) from 0.5 μg of total RNA using an Agilent Low RNA Input Fluorescent Linear Amplification Kit. Array hybridizations were carried out according to the manufacturer's instructions. Specifically, each array was hybridized with two samples, each of 0.75 μg labeled target cRNA, for 17 hours at 60°C. Data were acquired with an Agilent G2565BA scanner.

#### Microarray data analysis

Microarray experiments and data were managed and analyzed using a customized implementation of the BASE system [38]. The reliability and reproducibility of analyses was ensured by the use of triplicates in each experiment, the normalization of all 24 arrays to the median probe intensity level with background subtracted, and the use of well accepted and freely available software packages. The slide images were processed with FeatureExtraction v. 0.75 (Agilent). After filtering out saturated spots flagged by FeatureExtraction, we took an iterative approach to estimate non-specific hybridizations. For each of the three slides in a given experiment, we first calculated thresholds for background hybridizations with the 314 internal negative controls, as Average (median intensities - background) of negative controls] + 2 × standard deviation for both dyes. We then added to the 'non-hyb' (non-hybridizing) set only probes that showed below-threshold signals in at least five out of the six median intensities for the triplicates. Then iteratively, new thresholds for each slide were calculated and new non-hybridizing probes identified until there were none left. Probes that showed above-threshold signals in at least five out of the six median signals were labeled as the 'all-hyb' set. Observing a strong dye bias, we subjected the union of the 'all-hyb' sets for the 8 experiments (7,900 probes in total) to normalization for each slide. We chose the rank invariant method [39] for selecting non-differentially expressed genes and subsequently a loess fitness method for non-linear normalization using the identified invariant genes. After normalization, scaling procedures were applied to bring the variances of filtered and normalized expression values among the triplicates to the same variation level. Outliers were detected by a Grubb's test (*p *= 0.01) and flagged. The procedures were carried out using a MadScan BASE plug-in [40].

To estimate the number of transcripts for each tissue sample, we furthermore identified probes that showed below-threshold hybridizations for one dye but above-threshold hybridizations for the other. We required that all 3 dye intensities for the hybridizing samples to be over the 90 percentile of the median intensity of the 'all-hyb' set for it to be called 'present'. In the case of W23, which was used as the reference, at least five of the six dye intensities for a probe (from six hybridizations) must be larger than thresholds for it to be predicted as 'present' in W23 but 'absent' from the other two lines.

To assess differential expression, the Rank Products [41] method was used, which is a non-parametric testing against a random simulated background. It proves to be especially robust for our dataset given the presence of a large number of non-hybridizing probes and the single copy representation for most of the probes. To be more conservative, a slight modification to the algorithm was made which required all three log2 ratios to have the same sign ('+' for up-regulation and '-' for down-regulation) in order for a transcript to be picked as differentially expressed. The significance level was set to give a false discovery rate (FDR) of 5%. Hierarchical clustering of expressed genes was performed with EPCLUST [42], with correlation measure based distance and average linkage clustering methods. We used both normalized log2 ratios and log2 values of normalized absolute median intensities for clustering. When using absolute intensities, first we applied a linear regression to one test sample (either ND101/W23 or A619) based on normalized intensities of the common W23 reference tissue. Very good correlations were found between the W23 references for each of the four sets of experiments (all with r^2 ^> 0.90, data not shown). For W23 intensities, the mean was taken after scaling. Finally the values log_2_(scaled absolute intensity/median of the all-hyb set in the given tissue) were fed to the clusters.

### Array design and analysis - University of Arizona platform

To provide an additional level of confirmation and comparison between *in situ *synthesis and spotted arrays, six additional spotted arrays were utilized as part of a beta-testing study for the Maize Oligonucleotide Array Project (MOAP) [43]. This platform has approximately 58,000 spotted 70-mer oligonucleotide probes printed on two slides for each array. These were used as technical replicates for the experimental comparisons of the two anther stages between the W23 and A619 background already completed with the Agilent arrays. To minimize differences that might occur from separate cRNA preparations and to utilize valuable labeled sample, the protocol as recommended by MOAP was altered in the following ways. DNA probe immobilization was completed by placing each array DNA-side down over a 42°C water bath for 5 to 10 seconds. Slides were then immediately placed DNA-side up on a 70 to 80°C heat block and snap-dried for 3 to 10 seconds. DNA probes were then UV cross-linked to the slide at 65 mJ for 90 seconds using a UV Stratalinker 1800 (Stratagene, LA Jolle, CA, USA). Slides were incubated for 2 minutes in a 1% SDS bath, washed in a 95°C water bath for 2 minutes with gentle shaking, and finally placed in a water bath at room temperature to rinse briefly. Slides were centrifuged at 500 rpm for 5 minutes to dry, and stored in the dark at room temperature with desiccant until used. Hybridization methods were adopted from Agilent's protocol for processing oligoarrays except that 750 ng of each labeled cRNA sample was combined and hybridized to the slides at 55°C for 15 hours. Slide washing was done according to the MOAP protocol [43] and scanned in an Agilent G2565BA scanner.

The Arizona probe sequences were blasted against the set of EST contigs and singletons used to generate the 60-mers for the Agilent microarray. For each Arizona 70-mer, the top hit in the same orientation was selected among those with a minimum e-value of 1E-8, a minimum alignment of 68 bases, a maximum of 3 mismatches, and no gaps. There were 3,568 probe matches for slide A and 4,092 for slide B. The distance between each pair of probes was determined by comparing each Arizona probe's blast start position to the start position of the matching Agilent probe within the source EST contig or singleton. Scatter plots were generated using the basic plot function in R.

### Gene Ontology analysis

Because currently no maize GO project exists, we used the Blast2Go program [16] for our GO data mining. Blast2Go started with a Blastx similarity search (with e-value of 1E-10) against the *nr *NCBI protein database. Statistically significant matches were then assigned to each query, and GO annotations were mapped from known associations. To reduce errors we used GO annotations from the TIGR Maize Gene Index dataset if provided, which covered more than 2,000 hybridizing transcripts on the array. To assess significant over-representation of the GO terms we used Gossip [17], which takes an heuristic approach to control the family-wise error rate (FWER) as the multiple testing correction and outputs three *p* values: one for a single test, one adjusted *p* value to control the FWER, and one adjusted *p* value to control the FDR. For out tests, we required the *p* value for the single test to be less than 0.005 and the other two adjusted *p* values to be less than 0.1.

### Quantitative real-time PCR verification

DNase-treated RNA was reverse transcribed with poly-dT primer using a SuperScript III cDNA synthesis kit (Invitrogen), and stored at -20°C. Several reactions were pooled to avoid reaction-related variations. Primers were designed using Primer3 [44] and synthesized by Operon (Huntsville, AL, USA). Primer sequences are provided in Additional data file 6. All primers were tested to ensure amplification of single discrete bands with no primer-dimers. Melting curves were performed on the product to test if only a single product was amplified. Samples were also evaluated on a 2% agarose gel to confirm that a single product of the correct size was generated. The PCR products were purified from the gel and sequenced to verify their identities in some cases. Real-time PCR was carried out in a DNA Engine OPTICON2 (MJ Research, part of Bio-Rad, Hercules, CA, USA). Each reaction contained 1× buffer (with 2 mM MgCl_2_), 200 μM mixed dNTPs, 0.4 μU DyNAzyme II (MJ Research), 0.5× SYBR Green I (Molecular Probes, part of Invitrogen, Carlsbad, CA, USA), 0.25 μM of each primer, and about 12.5 ng cDNA in a final volume of 20 μl. Three replicates were performed for each sample plus template-free samples as negative controls. Cycling parameters consisted of an initial denaturation step at 94°C for 3 minutes, followed by 35 amplification cycles at 94°C for 15 seconds, 58°C for 15 seconds, and 72°C for 25 seconds. Fluorescence measurements were taken at the end of the annealing phase at 78°C, 82°C, and 86°C. The qRT-PCR data were analyzed using the 'mid-point' method, which calculates amplification efficiencies for each sample from its amplification profile [45]. Two internal standards were used for each tissue stage (Additional data file 6) and results averaged over all biological replications to reduce both systematic and biological variances.

## Additional data files

The following additional data are available with the online version of this paper. Additional data file 1 is a spreadsheet listing the putative stage-specific transcripts expressed in 1 mm anthers. Additional data file 2 is a spreadsheet listing the putative stage-specific transcripts expressed in 1.5 mm anthers. Additional data file 3 is a spreadsheet listing the putative stage-specific transcripts expressed in mature pollen. Additional data file 4 is a spreadsheet listing the putative stage-specific transcripts expressed in juvenile leaves. Additional data file 5 is a spreadsheet listing the transcripts with both sense and antisense probes hybridized. Additional data file 6 is a spreadsheet listing the primer sequences, putative gene product, and expression values for the qRT-PCR validation experiments. Additional data file 7 is a spreadsheet listing the transcripts potentially involved in cell cycle regulation and processes.

## Supplementary Material

Additional File 1 Putative stage-specific transcripts expressed in 1 mm anthers. Click here for file

Additional File 2 Putative stage-specific transcripts expressed in 1.5 mm anthers. Click here for file

Additional File 3 Putative stage-specific transcripts expressed in mature pollen. Click here for file

Additional File 4 Putative stage-specific transcripts expressed in juvenile leaves. Click here for file

Additional File 5 Transcripts with both sense and antisense probes hybridized. Click here for file

Additional File 6 Primer sequences, putative gene product, and expression values for the qRT-PCR validation experiments. Click here for file

Additional File 7 Transcripts potentially involved in cell cycle regulation and processes. Click here for file

## Figures and Tables

**Table 1:** Transcript expression analyzed by biological sample type

	A619			ND101/W23			W23	
	
	Total	Tissue-specific	Diff. exp (vs W23)	Total	Tissue-specific	Diff. exp (vs W23)	Total	Tissue-specific
	
Anther 1 mm	5,647	261	288	5,544	222	157	5,612	274
Anther 1.5 mm	5,714	201	278	5,564	155	163	5,690	214
Pollen	2,699	338	87	2,709	356	84	2,704	343
Juvenile leaf	5,873	967	320	5,810	971	237	5,770	909

Classes of hybridization are defined as follows: Total is the sum of all hybridizing transcripts; Tissue-specific probes exhibited positive hybridization signals in only one sample type, and differentially expressed (Diff. exp) transcripts were up- or down-regulated compared to the W23 reference samples in a particular tissue comparison. See Materials and methods for details.

**Table 2:** Significantly enriched GO terms in transcript groups

GO term	Number in test group	Number in reference group	GO description
**Anther-specific (667)**			
16,538	7	2	Cyclin-dependent protein kinase regulator activity
6,139	120	591	Nucleobase, nucleoside, nucleotide and nucleic acid metabolism
6,270	5	1	DNA replication initiation
			
**Pollen-specific (165)**			
30,599	9	10	Pectinesterase activity
4,857	10	23	Enzyme inhibitor activity
16,787	54	789	Hydrolase activity
31,410	39	513	Cytoplasmic vesicle
16,023	39	513	Cytoplasmic membrane-bound vesicle
16,789	10	38	Carboxylic ester hydrolase activity
4,553	12	62	Hydrolase activity, hydrolyzing O-glycosyl compounds
45,045	39	547	Secretory pathway
46,903	39	550	Secretion
16,798	12	69	Hydrolase activity, acting on glycosyl bonds
5,576	8	28	Extracellular region
42,545	4	2	Cell wall modification
6,810	57	1,063	Transport
51,234	57	1,065	Establishment of localization
3,779	6	16	Actin binding
51,179	57	1,068	Localization
8,092	7	25	Cytoskeletal protein binding
30,234	10	65	Enzyme regulator activity
45,330	3	1	Aspartyl esterase activity
7,010	10	68	Cytoskeleton organization and biogenesis
3,824	94	2,170	Catalytic activity
5,618	6	22	Cell wall
30,312	6	24	External encapsulating structure
8,150	136	3,596	Biological_process
30,036	5	16	Actin cytoskeleton organization and biogenesis
			
**Leaf-specific (490)**			
9,536	152	1,073	Plastid
19,825	5	3	Oxygen binding
			
**Expressed in all three tissue types (1,091)**			
12,505	29	43	Endomembrane system
			
**Differentially expressed, ND101/W23 pollen versus W23 pollen (47)**			
43,067	4	28	Regulation of programmed cell death
42,981	4	28	Regulation of apoptosis
6,916	3	12	Anti-apoptosis
43,069	3	14	Negative regulation of programmed cell death
43,066	3	14	Negative regulation of apoptosis
			
**Differentially expressed, ND101/W23 juvenile leaf versus W23 juvenile leaf (158)**			
16,491	22	304	Oxidoreductase activity
9,507	15	169	Chloroplast
9,579	7	40	Thylakoid

Only transcripts that showed detectable expression in all three lines were considered. The number of transcripts with GO terms assigned for each test group is shown in parentheses following the group description. The reference group comprises the rest of the transcriptome. The *p* values for each GO term are: *p *< 0.0005 for single testing, FWER adjusted *p *< 0.1 and FDR < 0.1. See Materials and methods for details.

**Table 3:** Analysis of antisense transcripts in the total transcriptome

		N (%/total)	Tissue-specific (%)	Differentially expressed
		
A619	Anther 1 mm	377 (6.6)	16 (4.2)	3
	Anther1.5 mm	435 (7.6)	39 (8.9)	5
	Pollen	388 (14.3)	44 (11.3)	2
	Juvenile leaf	644 (10.9)	214 (33.2)	4
				
ND101/W23	Anther 1 mm	372 (6.7)	17 (4.6)	0
	Anther1.5 mm	399 (7.2)	23 (5.8)	0
	Pollen	387 (14.3)	46 (11.9)	0
	Juvenile leaf	638 (11.0)	215 (33.7)	15
				
W23	Anther 1 mm	366 (6.5)	15 (4.1)	-
	Anther1.5 mm	433 (7.6)	40 (9.2)	-
	Pollen	387 (14.3)	44 (11.4)	-
	Juvenile leaf	642 (11.1)	215 (33.5)	-

**Table 4:** Significantly enriched GO terms in antisense transcripts

GO term	Number in test group	Number in reference group	GO description
Antisense (732)*			
50,874	23	51	Organismal physiological process
50,953	6	2	Sensory perception of light
7,601	6	2	Visual perception
5,816	8	6	Spindle pole body
5,815	12	17	Microtubule organizing center
922	12	17	Spindle pole
43,228	78	317	Non-membrane-bound organelle
43,232	78	317	Intracellular non-membrane-bound organelle
9,767	4	0	Photosynthetic electron transport
16,028	6	3	Rhabdomere
			
**Expressed sense-antisense pairs (120)**			
5,730	7	42	Nucleolus
6,575	7	44	Amino acid derivative metabolism
42,401	4	9	Biogenic amine biosynthesis
6,520	11	121	Amino acid metabolism
9,063	4	10	Amino acid catabolism
9,310	4	11	Amine catabolism
44,270	4	11	Nitrogen compound catabolism
9,072	5	23	Aromatic amino acid family metabolism
9,308	11	135	Amine metabolism

*The number of transcripts with GO terms assigned is shown in parentheses following the group description. See legend to Table 2 for details.
